# A Case Report of Idiopathic Intracranial Hypertension With Left-Sided Facial Spasm

**DOI:** 10.7759/cureus.63056

**Published:** 2024-06-24

**Authors:** Farman H Fatah, Zana A Mohammed

**Affiliations:** 1 General Physician, Shaheed Mala Yasein Health Center, University of Sulaymaniyah, Sulaymaniyah, IRQ; 2 Department of Neurology, Shar Hospital, University of Sulaymaniyah, Sulaymaniyah, IRQ

**Keywords:** hypertension, papilledema, hearing loss, facial spasm, idiopathic intracranial hypertension

## Abstract

Idiopathic intracranial hypertension (IIH) typically presents with increased intracranial pressure of an unknown origin. Facial spasms are an uncommon manifestation of IIH. We report a 56-year-old female patient displaying atypical IIH symptoms of left-sided facial spasm. Clinical examination and imaging confirmed the diagnosis of IIH, and the patient received treatment with acetazolamide. This case highlights the importance of considering IIH as a potential diagnosis in patients with facial spasms, especially when accompanied by other neurological symptoms. Early recognition, a high level of suspicion, and appropriate management are crucial for optimizing outcomes in IIH cases. Furthermore, collaboration among neurologists, neurosurgeons, radiologists, and ophthalmologists is essential for the comprehensive evaluation and management of IIH patients.

## Introduction

Idiopathic intracranial hypertension (IIH), also known as pseudotumor cerebri, presents a diagnostic challenge due to its unclear etiology and varied clinical manifestations. While it predominantly affects obese women of childbearing age, it can occur in individuals of any age or gender [1-3]. Common symptoms include headaches, pulsatile tinnitus, and transient visual obscurations [2,3]. However, IIH can also present with atypical symptoms or be associated with other neurological manifestations, complicating the diagnosis [2,3].

The pathophysiology of IIH remains elusive, with proposed mechanisms including impaired cerebrospinal fluid (CSF) absorption, elevated cerebral venous pressure, and hormonal dysregulation [4,5]. Diagnosis of IIH typically involves clinical evaluation and lumbar puncture, along with neuroimaging studies to exclude secondary causes of intracranial hypertension. Magnetic resonance imaging (MRI) of the brain is often performed to rule out structural lesions. While empty sella turcica is a common finding, MRI may also reveal other nonspecific findings or be entirely normal [1].

The management of IIH focuses on reducing intracranial pressure, alleviating symptoms, and preventing vision loss. Acetazolamide, a carbonic anhydrase inhibitor, is commonly used as first-line therapy to decrease CSF production and lower intracranial pressure [4,6,7]. Other treatment options include topiramate, which has been shown to be effective in some patients, and corticosteroids for acute exacerbations [8-10]. In refractory cases, surgical interventions such as ventricular shunt placement, lumboperitoneal shunt, and optic nerve sheath fenestration may be considered as last resort [11]. Weight loss and lifestyle modifications are also recommended to improve outcomes and reduce the risk of recurrence [12].

Despite advances in understanding and management, diagnosing and treating IIH can be challenging, particularly when the presentation is atypical [13,14] or when patients have concurrent medical conditions. Therefore, clinicians must maintain a high index of suspicion for IIH and consider a comprehensive differential diagnosis, including uncommon presentations and associated risk factors.

In this context, we present a case of IIH with a left-sided facial spasm, highlighting the importance of recognizing and appropriately managing atypical presentations of IIH. Through this case discussion, we aim to underscore the necessity of interdisciplinary collaboration and thorough evaluation in the diagnosis and management of IIH.

## Case presentation

A 56-year-old female with a body mass index (BMI) of 32.5 kg/m^2^ visited a neurologist at the request of an ophthalmologist for further investigation after a routine ophthalmologic check-up showed papilledema. She had suffered from left-sided facial spasm and was on regular Botox injections to the left side of her face to alleviate the spasm. At the time of the presentation, she did not report any associated visual changes or headaches. The patient’s past medical history was significant for hypertension, which was well-controlled with a combination of valsartan and amlodipine. She also had a history of type 2 diabetes mellitus, which she managed with metformin. There was no significant surgical history.

**Figure 1:**
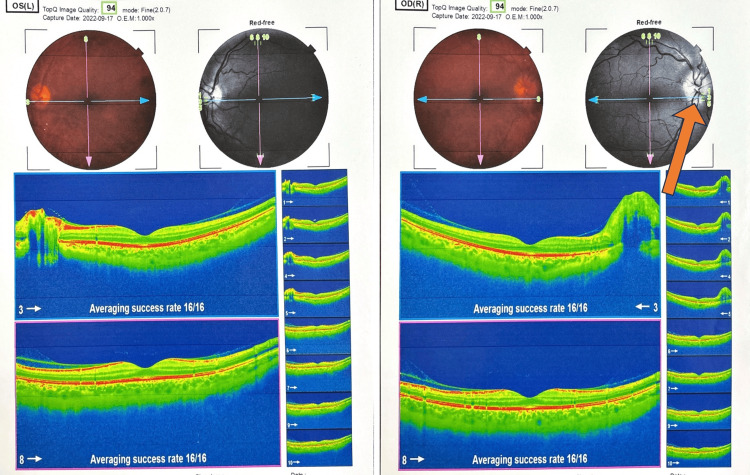
The optical coherence tomography (OCT) report of the left and right eyes The report shows optic disc edema on the right side.

**Figure 2:**
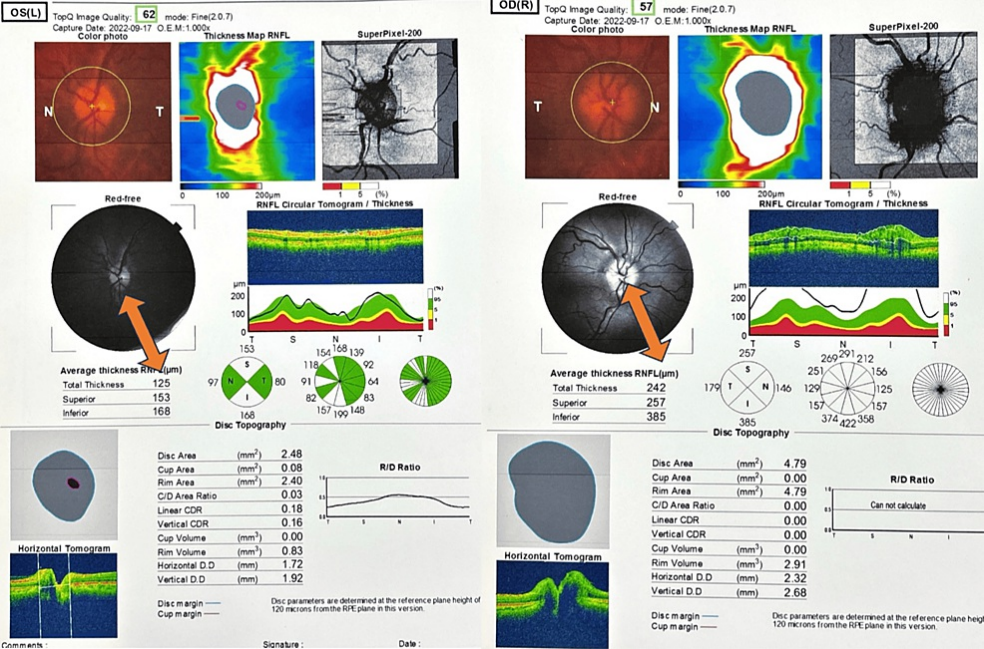
Left and right disc report with topography The average thickness of the retinal nerve fiber layer (RNFL) is increased on the right side with disc edema.

During the routine ophthalmologic examination, papilledema was incidentally detected on the fundoscopic examination. Subsequent optical coherence tomography (Figure 1) and topography (Figure 2) confirmed the presence of papilledema, prompting further investigation. A lumbar puncture revealed a CSF opening pressure of 215 mmH_2_O. The neurological examination was negative for other motor and sensory deficits, except for left hemi-facial spasm intermittently and left-sided sensorineural hearing loss (SNHL), which had occurred before the left hemi-facial spasm as a side effect of a medication for hypertension three years prior. This hearing deficit and facial spasm were subsequently improved after the initiation of acetazolamide treatment.

**Figure 3:**
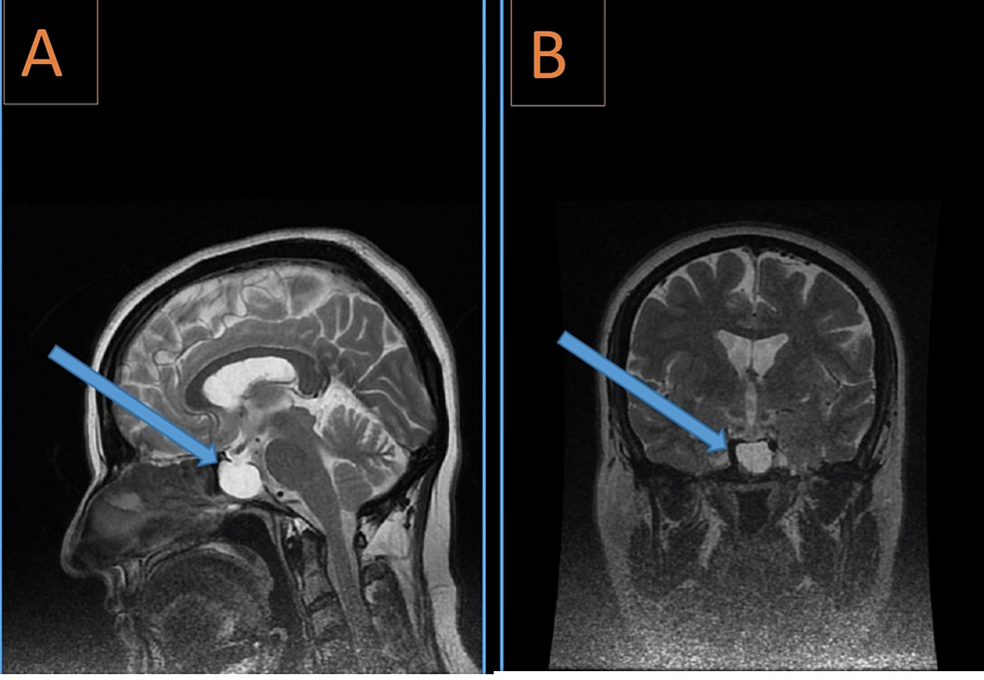
Magnetic resonance imaging of the brain Enlargement of sella turcica with empty sella on the (A) sagittal and (B) coronal views.

Further evaluation with an MRI of the brain revealed a small, negligible meningioma with a size of (11 mm × 7 mm) at the prepontine cistern on the left side. Additionally, MRI demonstrated an empty sella turcica with compression of the pituitary gland within the sella, consistent with findings commonly associated with IIH (Figure 3). Magnetic resonance venography (MRV) was also performed, which showed no evidence of venous sinus thrombosis or other abnormalities. In addition to facial spasms, the patient reported left-sided hearing loss, which had been present prior to the onset of facial spasms.

Following the initiation of acetazolamide at a dose of 250 mg once daily on the neurologist’s order, the patient experienced significant improvement in both her facial spasms and left-sided hearing loss over the course of several months. Following treatment initiation and improvement of symptoms, the patient opted to discontinue Botox injections at the affected site. Regular follow-up appointments were scheduled to monitor her clinical progress and adjust treatment as needed.

This case highlights the importance of a comprehensive diagnostic evaluation in patients presenting with facial spasms, even in the absence of typical IIH symptoms. Furthermore, collaboration among neurologists, neurosurgeons, radiologists, and ophthalmologists is essential for accurate diagnosis and optimal treatment outcomes in cases of IIH with associated facial spasms and hearing loss. Additionally, the patient’s history of hypertension and diabetes underscores the importance of considering comorbid conditions in the management of IIH.

## Discussion

The case presented underscores the complexity of diagnosing and managing IIH, particularly when atypical presentations are involved. This complexity emphasizes the need for a multidisciplinary approach and thorough evaluation in such cases. A comprehensive assessment is essential to exclude secondary causes of intracranial hypertension and to tailor treatment strategies effectively. A high index of suspicion is warranted for IIH, especially in patients with risk factors such as obesity, female gender, and young age.

Treatment of IIH aims to reduce intracranial pressure, alleviate symptoms, and prevent vision loss. Acetazolamide remains the cornerstone of pharmacotherapy, although other options, such as topiramate and corticosteroids, may be considered in certain cases [4,6-8,10]. Surgical options such as ventricular or lumboperitoneal shunts, in addition to optic nerve sheath fenestration, are viable treatments when medical therapy has failed [11]. Lifestyle modifications, including weight loss and dietary changes, are also crucial components of management [12]. However, atypical presentations, as seen in our case with left-sided facial spasm, can lead to diagnostic challenges and delays in appropriate management [2,3,13,14]. Therefore, clinicians must remain vigilant and consider IIH in the differential diagnosis of patients presenting with neurological symptoms, even when they are atypical.

## Conclusions

To conclude, our case underscores the importance of recognizing and managing atypical presentations of IIH. By promoting comprehensive evaluation through interdisciplinary collaboration among neurologists, neurosurgeons, radiologists, and ophthalmologists, we strive to improve outcomes and alleviate the burden of this challenging condition.
